# The Mediation Effect of Eudaimonic Well-Being in the Relationship Between Self-Determination and Somatic Symptoms

**DOI:** 10.3390/ijerph23060791

**Published:** 2026-06-12

**Authors:** Ivana Marcinko, Ana Kurtovic, Ana Babic Cikes

**Affiliations:** Department of Psychology, Faculty of Humanities and Social Studies in Osijek, University of J. J. Strossmayer, L. Jager 9, 31000 Osijek, Croatia; akurtovi@ffos.hr (A.K.); ababic@ffos.hr (A.B.C.)

**Keywords:** self-determination, controlling motives, eudaimonic well-being, negative affect, somatic symptoms, physical complaints

## Abstract

**Highlights:**

**Public health relevance—How does this work relate to a public health issue?**

- This work provides an understanding of how psychological aspects of functioning are associated with the presence of somatic symptoms.
- This work also helps determine the aetiological factors of somatic symptoms.

**Public health significance—Why is this work of significance to public health?**

- This study shows that the quality of motivation is important for the development of somatic symptoms.
- It also explains the mechanism through which self-determination gives rise to somatic symptoms.

**Public health implications—What are the key implications or messages for practitioners, policy makers and/or researchers in public health?**

- A holistic approach needs to be taken in order to understand and treat somatic symptoms.
- Successful treatments for somatisation need to address psychological factors to combat somatic symptoms with an unexplained medical cause.

**Abstract:**

The majority of somatic symptoms have unexplained medical causes, and it is claimed that psychological factors are important in the initiation and exacerbation of somatic complaints. This study, cross-sectional and correlational in nature, investigated the mediating role of eudaimonic well-being on the relationship between self-determination and somatic symptoms. Mediations were examined at both the whole-construct and component levels to better understand these relationships. A total of 486 participants took part in this study, comprising 403 females (82.9%) and 83 males (17.1%), with an age range of 18 to 36 years (*M* = 22, *SD* = 2.27). Self-determination, eudaimonic well-being, and somatic symptoms were measured using questionnaires. Mediations were tested at the construct and component levels using the PROCESS macro. The results show that eudaimonic well-being mediates the relationship between self-determination and somatic symptoms (*b* = −0.21, *SE* = 0.03, 95% *CI* = [−0.32, −0.10]). Component-level analyses reveal that the relationship between controlling motives and somatic symptoms is mediated by negative affect (*b* = 0.39, *SE* = 0.08, 95% *CI* [0.23, 0.56]). These findings identify the variables that may explain the origin of somatic symptoms, emphasising self-determination as a starting point and eudaimonic well-being as a mechanism by which motivational factors affect health outcomes.

## 1. Introduction

Somatic symptoms are physical complaints associated with different parts of the body and bodily functions. The most common somatic symptoms include nose, throat, and ear complaints; dizziness; shortness of breath; fatigue; gastrointestinal problems; musculoskeletal pains; and insomnia [1,2]. These problems are quite widespread among humans of all ages, and the prevalence of somatic complaints in the general population is estimated to range from 5% to 7.7% [3,4]. In the student population, the prevalence of somatic symptoms ranges from 5.7 to 84.4% [5,6], while among youth, it is approximated to be around 25% [7]. Somatisation is the tendency to experience somatic symptoms that have medically unexplained causes for which a person seeks medical attention. The manifestation of somatisation is usually initiated and exacerbated in the presence of psychological distress. Although not rooted in recognised physical dysfunctions, somatic symptoms and somatisation impose a great burden on the health system [8]. Up to 50% of doctor visits are due to somatic symptoms with no definable cause [9]. Thus, the origin of somatic symptoms, as well as somatisation, is still unknown.

Explanations for somatic symptoms and somatisation usually focus on psychological and social factors [10,11]. The predominant view of somatic symptoms as reflections of internal states and emotions is introduced by the psychoanalytic paradigm. Damasio [12,13] described so-called somatic markers as indicators of the brain’s responses to (consciously and unconsciously) processed stimuli [14]. According to him, somatic markers are manifestations of the bio-regulative processes that reflect one’s feelings [13]. Lumley and Schubiner [15] confirmed this premise by showing that unprocessed and unresolved emotional stress initiates and even worsens body symptoms.

The most common interpretation of somatic symptoms is that they represent repressed traumatic childhood experiences. Building on Freud’s concept of deferred action, Matthis [16] argued that early childhood experiences transform into somatic symptoms and that their full meaning is acquired later in life. The evidence for such an interpretation of physical symptoms comes from cross-sectional and longitudinal studies demonstrating that stressful experiences precede the development of somatic symptoms [17,18]. This is further confirmed by the fact that somatic symptoms commonly coexist with mental diseases such as anxiety and depression [19].

The alternative view is that somatic symptoms represent a maladaptive expression of psychic conflict. In other words, somatic symptoms are how the body communicates the presence of an internal problem. Marotti [20] argues that somatic symptoms carry an unconscious message bound to individual needs. For instance, burnout syndrome conveys the message that a person is unable to set boundaries in their life [21].

This stance, which holds that bodily complaints reflect internal struggles an individual faces and seeks to overcome, has been further investigated in contemporary approaches. One of the present-day theories, the self-determination theory (SDT) [22], suggests that the causes of somatic symptoms lie in the motivational aspects of human behaviour. This theory posits that the reason someone is doing a certain thing is a defining factor in either growth and thriving or decay and thwarting. As such, autonomy or self-determination represents motivational processes that are governed from within or from the authentic part of the self and are aligned with the fulfilment of one’s potential and needs. Thus, the higher the degree of self-determination, the greater the positive functioning and development. Within the theory, autonomy is also a psychological need that, if satisfied, ensures psychosocial prosperity. Motivational processes can also be rigid and controlling when initiated outside the self, setting in motion non-adaptive ways of functioning and ill health.

The theory proposes that for people with somatoform disorder, the self is experienced as ill, while their body is not part of the self [23]. This means that, for this population, the self is regulated by external pressures rather than by an autonomous segment of the self. In line with this, it is also expected that individuals motivated by an authentic self will have fewer somatic symptoms than those whose actions are driven by controlling motives. Some studies support this reasoning by providing evidence of the positive effect of self-regulation on the presence and severity of somatic symptoms. In such studies, autonomy has mainly been investigated as a psychological need. Olafsen et al. [24] demonstrated that workplace role conflict is linked to frustration of psychological needs, which in turn leads to burnout and somatic symptoms among workers in the health care system. Hatfield [25] investigated the association between psychological need satisfaction and denial with the severity of somatic symptoms among performing arts students. The results of his study show that the frustration of the need for autonomy and the need for competence contribute to the presence of somatic symptoms. The denial of psychological need satisfaction in the managerial work context increases somatic burden among its workers [11]. Finally, Sheikholeslamia and Arab-Moghaddam [26] determined a negative association between overall self-determination and somatic symptoms among Iranian students. Thus, indices show that a lower degree of self-determination contributes to the development of somatic symptoms.

Given that the literature provides limited insight into the role of self-determination in somatic symptoms, even less is known about how self-determination affects somatic complaints. Defining the mechanisms, or so-called mediators, through which self-determination contributes to somatic complaints would allow us to detect pathways through which motivated behaviour affects physical health outcomes. Drawing on the work of Miguelon and Vallerand [27], as well as up-to-date approaches that emphasise a holistic perspective on health, we argue that self-determined motivation through eudaimonic well-being contributes to somatic symptoms. The reason is that self-determination, as a starting point for activation of the human organism, sets multiple systems into motion synergistically [27]. During such activation, psychological and physical functioning are not distinct domains that work independently but form a unified whole. Self-determination, as a positive starting place of human activation, initiates a higher-order well-being state, i.e., eudaimonia, which arises from pursuits worth living for and personal growth, thereby contributing to physical health and fewer somatic symptoms. This reasoning is supported by findings that intrinsic goals are positively correlated with well-being [28], whereas extrinsic goals are negatively associated with well-being [29]. At the same time, studies from collectivistic cultures indicate a positive association between intrinsic and extrinsic goals and well-being [30,31], or no relationship between extrinsic motivation and well-being [32]. Kadzikowska-Wrzosek [33] came closer to testing relationships of interest by showing that positive and negative affect serve as mediators of the relationship between autonomy and somatic symptoms among breastfeeding women.

Thus, given the mind–body relationship mentioned in the explanation of the origin of somatic symptoms [27], this study aims to explore whether self-determination contributes to somatic symptoms via eudaimonic well-being. These relationships will be tested at the construct and component levels. Such an in-depth approach should provide a better understanding of the aetiology of somatic symptoms. Considering the literature, we hypothesise that:

**H1.**  *Eudaimonic well-being mediates the relationship between self-determination and somatic symptoms.*

**H2.**  *The relationship between autonomous motives and somatic complaints is mediated by positive affect.*

## 2. Materials and Methods

### 2.1. Participants and Procedure

Students from the University of J. J. Strossmayer in Osijek, Croatia, took part in the study. A representative sample of this population was extracted using a cluster sampling method. This process began by randomly selecting three Faculties (Faculties of Humanities and Social Sciences, Economics, and Teacher Education) from the pool of the existing ones within the University. In the next step, five departments from the selected faculties were randomly selected, followed by sampling individual classes until the desired number of participants was reached. Students were approached on a group level during their classes. Given that the classes students attended were compulsory and participation was anonymous, the response rate among the students present was high (above 95%). The research was cross-sectional. Prior to any assessment, participants received written and oral instructions on the purpose of the study and their rights, as well as basic instructions on how to respond to the questionnaire. Before the questionnaires, the participants answered questions about their health status. Results from those with chronic health conditions were excluded from the analyses, as they could affect the tested relationships.

After that, the goal assessment procedure took place, during which participants wrote down three goals. Then, they were instructed to complete the scales measuring self-determination and eudaimonic well-being while thinking about the chosen goals, and then complete the somatic symptom questionnaire.

In total, 511 participants completed the questionnaires, of whom 25 reported a chronic health condition; their results have therefore been excluded from further scrutiny. The data from 486 participants, of whom 403 (82.9%) were females, and 83 (17.1%) were males, were analysed. The participants were in the age range 18 to 36 (*M* = 22, *SD* = 2.27).

### 2.2. Measures

The presence of chronic illnesses was measured with the question: “Do you suffer from a chronic disease (e.g., asthma, diabetes, arthritis, etc.)?” to which the respondents answered by choosing between “yes/no” answers.

### 2.3. Self-Determination Measure

To assess the degree of self-determination (RAI) and the degree of autonomous and controlling motives for each participant, the Personal Goals Procedure and the Scale of Autonomous and Controlling Motives were used.

### 2.4. Personal Goals

The goal assessment procedure was based on the Personal Projects Model [34]. The instructions were as follows: “Goals are something that people think about, plan, implement through action, and sometimes (not always) bring to a conclusion and are successful in it. They may be more or less difficult to achieve, may take more or less time, and can be of varying interest or importance”. The participants were then told to list three of the most important goals they had been trying to achieve over the last six months and write them down. Once they did that, they were told to complete the autonomous/controlling motives scales, along with well-being scales linked to goals.

### 2.5. Scale of Autonomous/Controlling Motives (AM/CM) [35]

Autonomous and controlling motives were assessed by four items that measure four regulatory styles: intrinsic and identified (autonomous motivation), and introjected and external (controlling motivation). The item that measured the identified reason was “I aspire to this because I think it is really important to have a goal like this”, while the item that measured intrinsic reason was “I aspire to this because of the fun and pleasure that this activity gives me—my main reason is my interest in this experience”. The introjected reason was “I aspire to this goal because I will feel ashamed, guilty, or upset if I do not do it,” and the external reason was “I aspire to this goal because someone expects this of me or the situation imposes it on me”. The answers were given on a seven-point scale (from 1—not at all for this reason to 7—absolutely for this reason). The degree of self-determination or the relative autonomy index (RAI) associated with each goal is calculated using the formula 2 × intrinsic + identified − introjected − 2 × extrinsic [36]. The total degree of self-determination is calculated by summing the RAI scores across all three goals. Autonomous motives related to each goal are calculated by summing responses to items assessing identified and intrinsic reasons, while controlling motives are calculated by summing responses to items assessing introjected and extrinsic reasons. A single score of autonomous (controlling) motives is computed by summing responses from all autonomous (controlling) items obtained in relation to the three goals. In the reliability analysis of the self-determination scale, items measuring the controlled motive are reverse-scored. The internal reliability coefficient for the 12-item self-determination scale is 0.59. Furthermore, because the same statements were used three times when examining motives, the correlation between autonomous/controlling motives is determined across the three occasions. The correlations between the autonomous motives for the three goals range from 0.26 to 0.42, and those of the controlling motives range from 0.26 to 0.35.

### 2.6. Eudaimonic Well-Being

According to Veenhoven [37], a “good life,” i.e., eudaimonia, is the degree to which an individual judges their entire life favourably. Ryan et al. [38], on the other hand, define eudaimonia as a way of living, focusing on what is intrinsically worthwhile for a person. Following this logic, feelings that accompany intrinsic pursuits are not mere byproducts of such a lifestyle but reflect the degree to which one’s life has been worth living. So, defining eudaimonia in terms of the feelings that accompany worthwhile pursuits seems right. Given the analyses on the structure of subjective well-being, which indicate the presence of one general and three specific factors (positive affect, negative affect, and satisfaction with life) [39,40], and suggestions on how to measure well-being associated with human progress [41,42], the eudaimonic well-being measure is defined through affective and cognitive components. The affective component was measured using the Positive and Negative Affect Schedule, while the cognitive component was assessed using the single-item satisfaction-with-goal scale. These instruments were used three times, once for each goal. The total positive affect (PA) (inverted negative affect or satisfaction with a goal) score was obtained by summing the results across the scales for all three goals. The eudaimonic well-being measure is defined as the sum of PA scale scores, inverted NA scale scores, and a single-item satisfaction-with-goal scale associated with three goals. The internal reliability of the eudaimonic well-being scale across three measurement points ranges from 0.88 to 0.90.

### 2.7. Positive and Negative Affect Schedule (PANAS) [43]

PANAS measures two dimensions of emotion: positive affect and negative affect. Ten items measure positive emotions (e.g., careful, excited) and ten items measure negative emotions (e.g., irritable, frightened). Answers are given on a five-point scale ranging from 1 (very little or not at all) to 5 (extremely), bearing in mind the particular goal. The composite result for the PA component of eudaimonic well-being is calculated by summing the PA scale scores across all three goals. A higher result indicates a greater degree of PA associated with the goals. The composite result of the NA component of well-being is computed in the same manner. The internal reliability of the positive affect scale in this study ranges from 0.84 to 0.89, and that of the negative affect scale from 0.90 to 0.92.

### 2.8. Single-Item Satisfaction-with-Goal Measure

Given that the cognitive component of well-being is usually measured with multi-item or single-item scales assessing life satisfaction, whilst in this study the cognitive component assessed satisfaction with a goal, a single question usually used to define global life satisfaction [44] has been modified into “How satisfied are you in general with the achievement of this goal?” The answers are given on a seven-point Likert scale (1 = not satisfied at all to 7 = completely satisfied). The total satisfaction with goals score is calculated by summing the responses across all three goals. A higher result indicates higher satisfaction with goals.

### 2.9. Pennebaker of Limbic Languidness (PILL) [45]

PILL is a 54-item inventory aiming to measure the frequency of experiencing somatic symptoms such as headache, bad digestion, insomnia, or acne or pimples on the face. The instruction was to focus on the last six months, as that was the time frame participants were asked to consider when listing the goals they were pursuing. The answers are given on a five-point Likert scale (0 = never to 4 = very often). The result is calculated by summing across items; the higher the result, the greater the prevalence of somatic symptoms. The Cronbach’s alpha of the inventory in this study’s sample is 0.94.

### 2.10. Statistical Analysis

Statistical analyses were conducted using IBM SPSS Statistics Version 29 (Armonk, NY, USA). Descriptive analyses were conducted to assess the average scores and dispersion of the predictors, mediators, and criterion variables. Pearson correlation coefficients were calculated to assess relationships among the research variables in order to detect significant associations. The mediations between motives, eudaimonia, and somatic symptoms were examined using the PROCESS macro [46] model 4 with 5000 bootstrap samples. Prior to these analyses, the core assumptions were tested. No multicollinearity was detected; that is, all variance inflation factors were less than 1.5. There were no inflation cases since all Cook’s distances were significantly below 0.07 and, therefore, significantly below the cut-off value of 1. All residuals were normally distributed. Normal P-P Plot of regression standard residuals did not indicate a significant deviation from linearity. Scatterplots of standardised predicted values and standardised residuals did not indicate heteroscedasticity. The mediations were first tested at the level of overall constructs to determine whether any mediations exist. Once those were confirmed, the relationships were tested at the component level to better understand the pathways through which self-determination is linked to somatic symptoms. Thus, self-determination was assessed through the lens of autonomic and controlling motives, while the role of eudaimonic well-being was scrutinised in terms of the effects of positive and negative affect and goal satisfaction. All possible combinations of the self-determination and eudaimonic well-being components were examined. The significant findings are presented in the tables below, while the results of the other tests are presented in the Supplementary Materials.

## 3. Results

The descriptive statistics and correlations between the research variables are summarised in Table 1.

As can be seen, participants experienced a greater degree of autonomous motives than controlling ones when pursuing their goals. Also, the subjects reported a higher degree of positive emotions than negative ones. The experience of positive affect was quite high, while negative affect was moderately present. The presence of somatic symptoms among subjects was low.

The correlations revealed that the self-determination index [36] is negatively related to somatic symptoms. Surprisingly, controlling motives were positively associated with somatic symptoms, whereas autonomous motives were not significantly associated with the criteria. Eudaimonic well-being negatively correlated with the presence of somatic symptoms. Of all well-being components, only negative affect correlated with somatic complaints. The relationship between negative affect and somatic symptoms was positive.

The association between the RAI [36] or autonomous/controlling motives and well-being and its components was in the expected direction. The self-determination index [36] and autonomous motives were positively linked to eudaimonic well-being, positive affect, and satisfaction with goals. On the other hand, controlling motives were negatively related to eudaimonia and satisfaction with goals, while no association existed with PA.

Mediations were first tested at the level of entire constructs. The aim was to test the mediator effect of eudaimonic well-being on the relationship between self-determination and somatic symptoms. The mediation analysis summary is presented in Table 2.

As can be seen, the results indicated that self-determination significantly predicted eudaimonic well-being (*b* = 1.15, *p* < 0.001), which in turn predicted somatic symptoms (*b* = −0.18, *p* < 0.001). While the total effect of self-determination on somatic symptoms was significant (*b* = −0.25, *p* < 0.01), the direct effect was not (*b* = −0.04, *p* = 0.69). The bootstrapped analysis with 5000 samples revealed a significant indirect effect of self-determination on somatic symptoms through eudaimonic well-being (*b* = −0.21, *SE =* 0.03, 95% *CI* [−0.32, −0.10]), indicating full mediation. Thus, a higher self-determination index via higher eudaimonic well-being contributes to a lower degree of somatic symptoms. Having said that, the standardised coefficient (β) of the indirect effect is −0.09, indicating that the observed effect is small.

In the next step, mediations were examined at the component level. A series of Hayes’ [46] PROCESS macro analyses were undertaken to test whether autonomous or controlling motives are associated with somatic complaints through components of eudaimonic well-being. The predictors were autonomous or controlling motives, while mediators were positive and negative affect and satisfaction with goals. Of all analyses, mediation was confirmed in only one case, with negative affect as the mediator of the relationship between controlling motives and somatic symptoms. The results are presented in Table 3.

In this analysis, controlling motives predicted negative affect, (*b* = 1.05, *p* < 0.001), while negative affect predicted somatic symptoms, *b* = 0.37, *p* < 0.001. The total effect of controlling motives on somatic symptoms was also significant (*b* = 0.40, *p* < 0.001), while the direct effect was not (*b* = 0.01, *p* = 0.93), indicating full mediation. The indirect effect of controlling motives on somatic complaints via negative affect was confirmed to be significant (*b* = 39, *SE* = 0.08, 95% *CI* [0.23, 0.56]). The results show that a high degree of controlling motives via high negative affect is associated with a greater number of somatic complaints. However, the size of the standardised coefficient (β) for the indirect effect is 0.11, implying that the detected effect is rather small.

## 4. Discussion

This study investigated the role of psychological factors in the formation of somatic symptoms. The data revealed that high self-determination through high eudaimonia, a well-being based on self-actualisation and personal growth, is related to a low level of physical symptoms (*b* = 0.21, *S* = 0.03, 95% *CI* [−0.32, −0.10]). The analyses that scrutinised these relationships at the component level demonstrated that a high level of controlling motives through high negative affect predicts a greater degree of somatic symptoms (*b* = 0.39, *SE* = 0.08, 95% *CI* [0.23, 0.56]). Therefore, the research suggests that differences in the quality of motivation are linked to varying levels of well-being and, consequently, to diverse bodily somatic responses. So, although in research psychological and physical states are often, for practical and methodological reasons, viewed as separate constructs, they are not separate entities but a connected and integrated whole.

The positive association between self-determination, eudaimonic well-being, and fewer somatic symptoms could have been due to pursuits of self-congruent goals. Self-congruent goals are those that reflect the individual’s ongoing desires and drives for development and growth. The very selection of such a goal requires contact with the organismic evaluation process [47], the innate ability of a person to recognise and then select situations that stimulate personal growth. All goals that emerge from the organismic evaluation process reflect the individual’s interests, values, and desire for growth. Therefore, the efforts to achieve such goals lead to positive change within the organism, including both psychological and physical functioning. Research that supports this line of reasoning demonstrates the positive impact of self-congruent goals on well-being. Students who set self-congruent goals at the beginning of the academic year had greater well-being at the end of the second semester [48]. Self-congruence of goals is associated with well-being across cultures, regardless of cultural norms or societal values [49].

As some studies show that low-concordant goals are accompanied by low energy and distress [50], it is hypothesised that this may explain the findings. It is believed that the common denominator for low self-determination, low eudaimonia, and somatic complaints is ego depletion [51] or low vitality [52]. Both constructs refer to the low level of energy available to the self. This energy is greater when a person acts in accordance with themselves, that is, when they are autonomous. On the contrary, if a person’s behaviour feels controlled, this energy is depleted. Research has demonstrated that controlling situations lead to significant energy loss, negative emotions, and ill health compared with autonomously supportive circumstances [53]. Ryan et al. [54] conducted a series of experimental studies in which instructions were manipulated to create a context of autonomous or controlling motivation, and the after-effects of each were observed. The results showed that controlled forms of motivation contributed to tension and the perception of pressure, while autonomous motivation was associated with interest and satisfaction. Similarly, research by Sansone, Sachau, and Weir [55] suggests that motivated behaviours can lead to two possible outcomes. One involves an optimal mood, a state of high energy, interest, and excitement (i.e., health), while the other creates anxiety, frustration, and anger (i.e., illness). Thus, controlling motivation may represent a fertile ground for a ripple effect of declining eudaimonic well-being followed by somatic complaints.

Another possible explanation for the low self-determination–high somatic symptom relationship is seeing somatic symptoms as a reflection of psychic tensions and unprocessed emotions or trauma. Research indicates that somatic symptoms represent an unconscious message bound to the individual’s needs [20] and that subconscious emotions may not only initiate but worsen somatisation [15]. If controlled motivation depletes a person of deeply held wants and needs, it is expected that control-motivated pursuits contribute to the formation of somatic symptoms. Oliver, Markland, Hardy, and Petherick’s [56] work confirmed this by demonstrating that controlled motivation is associated with a greater prevalence of unspoken negative emotions. The inner speech of people whose behaviours feel controlled is characterised by a high degree of negative emotions and swear words. Additionally, longitudinal studies confirm that internally experienced negative emotions negatively affect health status, whereas externally expressed negative emotions do not [57].

This study also provides scrutiny of assessed relationships through component-level analyses. It has been shown that there is a distinctive negative pathway between motives and eudaimonic components that give rise to somatic symptoms. The study suggests the developmental trajectory of somatic symptoms. Previous work on the relationships between motives and health emphasises the importance of the interplay between autonomous motives and positive affect [27], while this study demonstrates that controlling motives only through one component of eudaimonia—a negative affect—is associated with somatic complaints. So, contrary to earlier findings, as well as our hypothesis, autonomous motives did not predict somatic symptoms, nor did positive affect mediate the relationship between motives and somatic symptoms. The findings suggest that the controlling type of motivation creates an internal milieu that is unnatural and stressful for an individual, which is then linked to negative emotion and, consequently, somatic complaints. Earlier studies have shown that somatisation is associated not only with psychological pressure but also with physiological processes that convey the body’s stress response. Masari [58] demonstrated that the presence of stress and somatic symptoms is particularly strong among extrinsically motivated people compared with those motivated by other types of motives. On the other hand, Steel et al. [59] have shown that controlling motivation is linked to an increased acute cortisol response, commonly seen in the presence of threat [60], whereas during autonomously motivated behaviours, this stress response is reduced.

Generally speaking, our study’s results indicate that the relationship between motives and somatic symptoms is multi-layered. Motives are linked to somatisation through a higher-level sense of eudaimonia, and also through a lower-level sense, i.e., its components. Also, despite the literature suggesting the importance of positive affect for optimal health [61,62], this study suggests that the role of negative affect in the aetiology of somatic complaints is greater. Having said that, one must be cautious with such a conclusion, as the well-being component, which explained somatic symptoms in this study, may have been extracted in the analyses due to the prevalence of female participants in the sample. Cross-cultural data [63] demonstrates that females, as opposed to males, tend to have higher neuroticism, which predisposes them to frequent experiences of negative affect, anxiety, and threat while perceiving ordinary events as overwhelming. So, for a predominantly female sample, it is not unusual that negative affect, rather than positive, was associated with somatic symptoms. Thus, in the event of a different male-to-female ratio in the sample, a different well-being component could prove pivotal for somatic symptoms.

Another topic that needs to be addressed is that while the majority of research points towards high levels of autonomous motives as crucial for optimal health [64], this study suggests that low levels of controlling motives are more important for a low presence of somatic symptoms. Here, gender differences may also have played a role, as research shows that males tend to experience greater self-determination across domains than females [65]. So, the inclination towards controlling ways of functioning may be common among females who naturally gravitate towards negative affect [64].

We also cannot rule out the possibility that the discrepancy between our findings and those of earlier studies arose from the culture in which this research was conducted. Self-determination theory indicates that self-determination of behaviour depends on various proximal and distal factors [66]. One of the proximal factors that influences the degree of self-determination is the environment in which an individual lives. Cultural values have not been examined in the study’s sample; however, drawing on others’ work, we hypothesise about the participants’ societal beliefs. On Hofstede’s individualism dimension, which indicates the extent to which individuals are oriented towards looking after themselves or others, Croatia gravitates towards collectivistic values, meaning that behaviour is predominantly driven by group needs and acceptance [67]. On the masculinity–femininity dimension, which indicates the degree to which a society’s members are assertive or concerned about others’ feelings, Croatia’s score points to predominantly feminine tendencies [68]. Under such a hierarchy of values, Croatian people, as in all other collectivistic societies, value traits such as interrelatedness, obedience, and submission [69], or in other words, they are part of the environment which imposes control on its members. Under such circumstances, practicing autonomous regulation would, in our view, seem less likely because it would be seen as socially unwelcome behaviour, so positive psychological and physical functioning would depend not on experienced autonomy but on a lower level of imposed control. We believe that under the pressure of a controlling cultural framework, a person will acquire self-determination, well-being, and fewer somatic symptoms if the control asserted by the social context is lower.

In addition, collectivistic as opposed to individualistic cultures view the expression of somatic symptoms as an appropriate way to convey emotional tension [70,71], which also may offer some insight into why a high degree of controlling motives, rather than a low degree of autonomous ones, was associated with somatic complaints. It is speculated that for subjects in this study, being rooted in collectivistic values meant that somatic symptoms were an acceptable way to communicate internal conflicts and the strain caused by the controlling motivation tied to their goal pursuits.

The active role of controlling motives and negative affect in the formation of somatic symptoms in this study supports the fundamental premises of positive psychology, which posits that factors that give rise to optimal health differ from those responsible for detrimental health [72]. The salutogenic model of health [73], being interested in the correlates of health, assumes that autonomous motives and positive affect are central to optimal functioning, while the pathogenic model, where the focus is on predictors of illness, posits that risk factors, such as controlling motives and negative affect, in our case, precipitate somatic symptoms. Thus, the revelation of the distinct pathway linking negative psychological assets to the presence of somatic symptoms in this study supports the pathogenetic model of health. Such a finding is consistent with earlier studies, which demonstrate a clear distinction between correlates of healthy and detrimental functioning [74,75].

### Limitations

This study was cross-sectional and correlational, so our ability to draw conclusions about the causality and directionality of the tested relationships is quite limited. Given previous research, it is less likely that the tested relationships go in the opposite direction (somatic symptoms predicting self-determination). However, to be certain, a longitudinal approach would allow us to test the true nature of the relationships. The study’s sample consisted of students, so the findings cannot be generalised to other groups. Research with different populations in terms of age, education, or different male-to-female ratios may prompt different results. For example, because self-determination is more pronounced among older people [76], it is assumed that autonomous motives rather than controlling ones may predict somatic symptoms in research with this population. Eudaimonia, on the other hand, is more salient among educated people [77], so including a greater number of those with higher education or elementary schooling could yield different outcomes. As said earlier, testing the relationships in a predominantly female sample could have identified controlling motives and negative affect as salient correlates of somatic complaints, given women’s tendencies toward high neuroticism [63]. Thus, having a more balanced male–female ratio in the sample may prompt different results. Further, the exclusion of the participants with chronic conditions does not rule out the possibility that individuals with mental disorders or acute health problems in the sample have not influenced the results. To avoid this, the question(s) assessing medical conditions need to be more carefully formulated to exclude all these groups. Somatic symptoms were measured as general self-reported complaints rather than clinically evaluated medically unexplained symptoms, which represents another shortcoming of this study. The lack of a traditional operationalization of eudaimonia through dimensions such as personal growth, autonomy, or life purpose limits the comparison of our results with those of previous studies. The implementation of self-reported questionnaires is another limitation of our findings. The data in the study could have been subject to self-report bias and common method variance. To avoid these problems, alternative measures of somatic symptoms may be a better choice (e.g., objective measures from medical records) or multiple time points if self-reported measures are still preferred. The internal validity of the self-determination index scale (α = 0.59) is seriously problematic, raising doubts about whether the findings were falsely positive. The implementation of a single-item scale to measure satisfaction with goal progress could, due to its low reliability, weaken the mediator’s construct validity; therefore, future studies should consider using multi-item scales to measure this component of eudaimonic well-being. Performing multiple tests at the component level does not rule out the possibility that the results are due to a Type I error rather than reflecting the true relationships between variables. Finally, although the indirect effects of motives on somatic symptoms were statistically significant, the observed effects were small, suggesting that mediators have a minor role for somatic symptoms in the real world. All of the above suggest that the findings cannot be taken at face value and require further examination in future studies.

## 5. Conclusions

The aim of this study was to investigate the mediation effect of eudaimonic well-being in the relationship between self-determination and somatic symptoms. The study findings show that higher self-determination via greater eudaimonic well-being is associated with fewer somatic symptoms. Analysing these relationships at the component level provided further insight. The more pronounced the controlling motives that accompany goal-pursuing behaviour, the greater the negative affect, which leads to a higher degree of somatic symptoms. The research reveals that the level of experienced control, tied to goal-directed behaviour, determines the quality of psychological functioning and the severity of bodily complaints.

## Figures and Tables

**Table 1:** The descriptive statistics and correlations for the study variables.

Variable	*M*	*SD*	Min/Max	1	2	3	4	5	6	7	8
1. Eudaimonic well-being	236.31	32.39	63–321								
2. PA (PANAS)	108.73	18.09	30–150	0.76 **							
3. NA (PANAS)	68.26	20.84	30–150	−0.81 **	−0.24 **						
4. Satisfaction with goals	15.83	3.32	3–21	0.61 **	0.53 **	−0.34 **					
5. Self-determination index	10.51	13.04	−36–36	0.46 **	0.32 **	−0.39 **	0.31 **				
6. Autonomous motives AM/CM	34.33	5.60	6–42	0.32 **	0.44 **	−0.07	0.28 **	0.55 **			
7. Controlling motives AM/CM	20.58	8.52	6–42	−0.33 **	−0.07	0.43 **	−0.17 **	−0.61 **	0.07		
8. Somatic symptoms (PILL)	66.62	28.25	0–216	−0.22 **	−0.05	0.27 **	−0.11 *	−0.12 **	0.00	0.12 **	

*M*—mean; *SD*—standard deviation; Min/Max—minimum and maximum values; PANAS—Positive and Negative Affect Schedule; AM/CM—scale of autonomous/controlling motives; PILL—Pennebaker of Limbic Languidness; ** *p* < 0.01, * *p* < 0.05.

**Table 2:** The mediating role of eudaimonic well-being in the relationship between self-determination and somatic symptoms.

Effect Type	Path	Coefficient (*b*)	*SE*	*t*	*p*	95% *CI* [LL, UL]
**Direct Effects**						
Self-determination ⟶ eudaimonia	*a*	1.15	0.09	11.59	<0.001	[0.95, 1.34]
Eudaimonia ⟶ somatic symptoms	*b*	−0.18	0.04	−4.29	<0.001	[−0.26, −0.09]
Self-determination ⟶ somatic symptoms	*c*′	−0.04	0.10	−0.38	0.69	[−0.24, 0.17]
**Total Effect**						
Self-determination ⟶ somatic symptoms	*c*	−0.25	0.09	−2.59	<0.01	[−0.43, −0.06]
**Indirect Effect**						
Self-determination ⟶ eudaimonia ⟶ somatic symptoms	*a* × *b*	−0.21	0.03	-		[−0.32, −0.10]

*b*—unstandardized coefficient; *SE*—standard error; *t*—test statistic; *p*—statistical significance level; *CI*—confidence interval; LL—lower-level confidence interval; UL—upper-level confidence interval.

**Table 3:** The mediating role of negative affect in the relationship between controlling motives and somatic symptoms.

Effect Type	Path	Coefficient (*b*)	*SE*	*t*	*p*	95% *CI* [LL, UL]
**Direct effects**						
Controlling motives ⟶ negative affect	*a*	1.05	0.09	10.79	<0.001	[0.86, 1.24]
Negative affect ⟶ somatic symptoms	*b*	0.37	0.06	5.81	<0.001	[0.24, 0.50]
Controlling motives ⟶ somatic symptoms	*c*′	0.01	0.15	0.07	0.93	[−0.29, 0.32]
**Total effect**						
Controlling motives ⟶ somatic symptoms	*c*	0.40	0.14	2.77	<0.01	[0.11, 0.69]
**Indirect effect**						
Controlling motives ⟶ negative affect ⟶ somatic symptoms	*a* × *b*	0.39	0.08	-		[0.23, 0.56]

*b*—unstandardized coefficient; *SE*—standard error; *t*—test statistic; *p*—statistical significance level; *CI*—confidence interval; LL—lower-level confidence interval; UL—upper-level confidence interval.

## Data Availability

The data presented in this study are openly available in the Figshare database at https://doi.org/10.6084/m9.figshare.24548845 (12 November 2023).

## Supplementary Materials

The following supporting information can be downloaded at: https://www.mdpi.com/article/10.3390/ijerph23060791/s1, Table S1. The results of the mediation analyses testing the mediating role of positive affect on the relationship between autonomous motives and somatic symptoms. Table S2. The results of the mediation analyses testing the mediating role of negative affect on the relationship between autonomous motives and somatic symptoms. Table S3. The results of the mediation analyses testing the mediating role of satisfaction with goals on the relationship between autonomous motives and somatic symptoms. Table S4. The results of the mediation analyses testing the mediating role of positive affect on the relationship between controlling motives and somatic symptoms. Table S5. The results of the mediation analyses testing the mediating role of satisfaction with goals on the relationship between controlling motives and somatic symptoms.
