# Supplementary material for: The Mediation Effect of Eudaimonic Well-Being in the Relationship Between Self-Determination and Somatic Symptoms
Source: Int J Environ Res Public Health. 2026 Jun 12;23(6):791. doi: 10.3390/ijerph23060791 (PMC13299252; doi:10.3390/ijerph23060791)
Supplement: Supplementary file 1 [file ijerph-23-00791-s001.zip › ijerph-4301408-supplementary.pdf]

Supplementary materials

The additional models tested during component-level analyses

Table S1. The results of the mediation analyses testing the mediating role of positive affect on the relationship between autonomous motives and somatic symptoms

Effect type	Path	Coefficient (*b*)	*SE*	*t*	*p*	95% *CI* [LL, UL]
**Direct effects**						
Autonomous motives → positive affect	*a*	1.42	0.12	11.03	<0.001	[1.17, 1.67]
Positive affect → somatic symptoms	*b*	-0.10	0.77	-1.33	0.18	[-0.25, 0.04]
Autonomous motives → somatic symptoms	*c'*	0.12	0.24	0.50	0.61	[-0.36, 0.61]
**Total effect**						
Autonomous motives → somatic symptoms	*c*	-0.02	0.22	-0.09	0.92	[-0.46, 0.42]
**Indirect effect**						
Autonomous motives → positive affect → somatic symptoms	*a* × *b*	-0.14	0.11	-		[-0.36, 0.08]

b—unstandardized coefficient; *SE*—standard error; *t*— test statistic; *p*—statistical significance level; *CI*—confidence interval; LL—lower-level confidence interval; UL—upper-level confidence interval

Table S2. The results of the mediation analyses testing the mediating role of negative affect on the relationship between autonomous motives and somatic symptoms

Effect type	Path	Coefficient (*b*)	*SE*	*t*	*p*	95% *CI* [LL, UL]
**Direct effects**						
Autonomous motives → negative affect	*a*	-0.26	0.16	-1.61	0.11	[-0.58, 0.05]
Negative affect → somatic symptoms	*b*	0.37	0.05	6.49	<0.001	[0.26, 0.49]
Autonomous motives → somatic symptoms	*c'*	0.08	0.21	0.37	0.71	[-0.34, 0.50]
**Total effect**						
Autonomous motives → somatic symptoms	*c*	-0.02	0.22	-0.09	0.93	[-0.46, 0.42]
**Indirect effect**						
Autonomous motives → negative affect → somatic symptoms	*a* × *b*	-0.10	0.07	-		[-0.25, 0.02]

b—unstandardized coefficient; *SE*—standard error; *t*— test statistic; *p*—statistical significance level; *CI*—confidence interval; LL—lower-level confidence interval; UL—upper-level confidence interval

Table S3. The results of the mediation analyses testing the mediating role of satisfaction with goals on the relationship between autonomous motives and somatic symptoms

Effect type	Path	Coefficient (*b*)	*SE*	*t*	*p*	95% *CI* [LL, UL]
**Direct effects**						
Autonomous motives → satisfaction with goals	*a*	0.17	0.02	6.63	<0.001	[0.12, 0.22]
Satisfaction with goal → somatic symptoms	*b*	-0.96	0.38	-2.53	<0.01	[-1.71, -0.21]
Autonomous motives →somatic symptoms	*c'*	0.14	0.23	0.62	0.53	[-0.31, 0.60]
**Total effect**						
Autonomous motives → somatic symptoms	*c*	-0.02	0.22	-0.09	0.92	[-0.46, 0.42]
**Indirect effect**						
Autonomous motives → satisfaction with goals → somatic symptoms	*a* × *b*	-0.16	0.07	-		[-0.32, 0.02]

b—unstandardized coefficient; *SE*—standard error; *t*— test statistic; *p*—statistical significance level; *CI*—confidence interval; LL—lower-level confidence interval; UL—upper-level confidence interval

Table S4. The results of the mediation analyses testing the mediating role of positive affect on the relationship between controlling motives and somatic symptoms

Effect type	Path	Coefficient (*b*)	*SE*	*t*	*p*	95% *CI* [LL, UL]
**Direct effects**						
Controlling motives → positive affect	*a*	-0.15	0.09	-1.64	0.09	[-0.34, 0.02]
Positive affect → somatic symptoms	*b*	-0.07	0.06	-1.04	0.29	[-0.20, 0.06]
Controlling motives → somatic symptoms	*c'*	0.39	0.14	2.69	<0.01	[0.10, 0.68]
**Total effect**						
Controlling motives → somatic symptoms	*c*	0.40	0.14	2.77	<0.01	[0.11, 0.69]
**Indirect effect**						
Controlling motives → positive affect → somatic symptoms	*a* × *b*	0.01	0.01	-		[-0.01, 0.04]

b—unstandardized coefficient; SE—standard error; *t*— test statistic; *p*—statistical significance level; CI—confidence interval; LL—lower-level confidence interval; UL—upper-level confidence interval

Table S5. The results of the mediation analyses testing the mediating role of satisfaction with goals on the relationship between controlling motives and somatic symptoms

Effect type	Path	Coefficient (*b*)	*SE*	*t*	*p*	95% *CI* [LL, UL]
**Direct effects**						
Controlling motives → satisfaction with goals	*a*	-0.06	0.01	-3.88	<0.001	[-0.10, -0.03]
Satisfaction with goals → somatic symptoms	*b*	-0.74	0.37	-2.02	0.04	[-1.47, -0.02]
Controlling motives → somatic symptoms	*c'*	0.35	0.14	2.40	<0.01	[0.06, 0.64]
**Total effect**						
Controlling motives → somatic symptoms	*c*	0.40	0.14	2.77	<0.01	[0.11, 0.66]
**Indirect effect**						
Controlling motives → satisfaction with goals → somatic symptoms	*a* × *b*	0.05	0.02	-		[0.00, 0.11]

b—unstandardized coefficient; *SE*—standard error; *t*— test statistic; *p*—statistical significance level; *CI*—confidence interval; LL—lower-level confidence interval; UL—upper-level confidence interval
